# Dermal Alpha‐Synuclein Aggregation in Seed Amplification Assays for Parkinson's Disease Subtype Differentiation

**DOI:** 10.1111/ene.70453

**Published:** 2025-11-28

**Authors:** Magdalena Vieregge, Anastasia Kuzkina, Annette Janzen, Wolfgang H. Oertel, Michael Sommerauer, Jens Volkmann, Kathrin Doppler

**Affiliations:** ^1^ Department of Neurology University Hospital Würzburg Würzburg Germany; ^2^ Ann Romney Center for Neurologic Diseases, Brigham and Women's Hospital and Harvard Medical School Boston Massachusetts USA; ^3^ Department of Neurology Philipps University Marburg Marburg Germany; ^4^ Helmholtz Center for Health and Environment, Institute for Neurogenomics München‐Neuherberg Germany; ^5^ Cognitive Neuroscience, Institute for Neuroscience and Medicine (INM‐3) Research Centre Juelich Juelich Germany; ^6^ Department of Neurology, Faculty of Medicine and University Hospital Cologne, University of Cologne Cologne Germany; ^7^ German Centre for Neurodegenerative Diseases (DZNE), Bonn Germany; ^8^ Center of Neurology, Department of Parkinson, Sleep and Movement Disorders University of Bonn Bonn Germany

**Keywords:** alpha‐synuclein, Parkinson's disease, REM sleep behavior disorder, seeding amplification assay, skin biopsy

## Abstract

**Background:**

Skin biopsies and seed amplification assays (SAA) provide a sensitive and potentially quantitative method to detect alpha‐synuclein (a‐syn) aggregation in peripheral nerve fibers in Parkinson's disease (PD). Relating to the previously published hypothesis that PD may either originate in the peripheral (body‐first) or central (brain‐first) nervous system, we investigated whether patients with clinical features that have been reported to be associated with a suspected body‐first subtype of PD exhibit higher levels of a‐syn aggregation in dermal nerve fibers compared to those without these features. Patients with isolated REM sleep behavior disorder (iRBD) representing a suspected premotor stage of body‐first PD were studied in comparison to the PD cohort.

**Methods:**

Patients were categorized on the basis of clinical features, and SAA parameters such as lag time, number of positive curves, and titers were analyzed and correlated with clinical features.

**Results:**

Although patients with clinical features of suspected body‐first PD showed slightly higher titers, significant differences were mainly observed between iRBD patients and PD patients.

**Conclusions:**

Our data suggest that widespread α‐syn aggregation in advanced PD limits the use of SAA in skin biopsies for subtype differentiation.

## Graphical abstract

Dermal alpha‐synuclein seed amplification assay of patients with suspected body‐first, brain‐first Parkinson's disease and isolated REM sleep behavior disorder was performed and quantified. Titers of body‐first patients were slightly higher compared to brain‐first PD, and were higher in iRBD compared to PD patients, but SAA‐based subtype differentiation was limited by widespread dermal alpha‐synuclein aggregation in advanced PD.

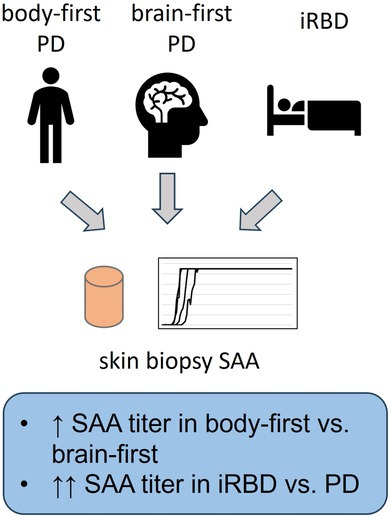

## 1 Introduction

Skin biopsies are an easily accessible tissue to investigate alpha‐synuclein (a‐syn) aggregation in the peripheral autonomic nervous system of patients with Parkinson's disease (PD) [1]. In the last few years, seed amplification assays (SAA) were established as a sensitive and specific tool to detect even small amounts of a‐syn aggregates [2, 3]. Quantification of a‐syn seeding is difficult because of the non‐linearity of the SAA reaction, the stochastic nature of aggregation, and even small variations of experimental conditions may affect SAA kinetics [4]. Lag time, the number of positive replicates, and titration of samples were proposed as possible quantitative measures in previous studies, as correlation with disease progression markers could be demonstrated [5, 6]. Regarding the previously proposed model of a brain‐ and body‐first subtype of PD, which proposes that a‐syn aggregation either propagates from (brain‐first) or to (body‐first) the central nervous system [7], quantification of peripheral a‐syn aggregation may be of interest to differentiate between these subtypes. In previous studies, patients with isolated REM sleep behavior disorder (iRBD), which is supposed to be a premotor stage of body‐first PD [8, 9] were more frequently positive by dermal SAA than PD patients, thus supporting the idea of more abundant a‐syn aggregation in peripheral autonomic nerve fibers in this subgroup [10].

In the present study, we categorized a mixed cohort of PD patients comprising various stages and disease durations according to suspected clinical features of brain‐ and body‐first subtypes. We used different quantitative parameters of SAA to investigate whether dermal SAA may allow us to identify body‐first subtypes on the basis of their α‐syn load in peripheral nerves. A cohort of iRBD patients that is supposed to represent a reliable cohort of premotor body‐first PD served as a comparison.

## 2 Material and Methods

### 2.1 Study Participants

Skin samples of patients with a diagnosis of PD (*n* = 39) according to the MDS clinical diagnostic criteria [11], with video‐polysomnographically confirmed iRBD (*n* = 15) [12] and controls (*n* = 7) without any signs or symptoms of a movement disorder who had been recruited at the University Hospitals Würzburg (PD patients), Cologne and Marburg (iRBD samples) for a previous study [10] were included. All participants had given written informed consent, and the study was approved by the ethics committees of the University of Würzburg, Cologne and Marburg.

Clinical data were collected from the patients' records; iRBD screening questions, Montreal Cognitive Assessment test (MoCA) and the non‐motor symptom score (NMSS) had been conducted as part of our previous study [10].

The following clinical features that are suspected indicators of body‐ or brain‐first PD [7, 9] were compared: young or old age of onset (< 51 years, > 53 years), RBD screening question negative/positive, tremor‐dominant or hypokinetic subtype, slow or rapid progression (< 0.167 H&Y/year, > 0.2), MoCA test (< 25 > 26 points), NMSS (< 34, > 39 points). For an overall assessment of the suspected subtype, patients were categorized into the subtype in which they showed the most features resulting in 20 patients with suspected body‐first PD and 19 patients with suspected brain‐first subtype.

Lysates of skin samples had been prepared for a previous study [10] and were stored at −80°C until use. One skin sample per patient was studied. Skin biopsies from the neck (C7, *n* = 26) or upper leg (*n* = 13) of PD patients and from the neck of all iRBD patients were included. As we did not find a significant difference in SAA data between the two biopsy sites, data from both sites were analyzed together.

### 2.2 Seed Amplification Assay (SAA)

Six to eight silica beads per well were first added to a black 96‐well plate (Nunc, ThermoFisher, USA). SAA buffer, consisting of 0.1 M PIPES pH 6.5, 500 mM NaCl as well as 20 μM Thioflavin and 1 mg/mL recombinant C‐terminal his‐tagged a‐syn (as described in [3]) was prepared.

Skin lysates were diluted in DPBS with N2 supplement with a standard dilution of 1:10. For determining titers, dilutions of 1:10, 1:50, 1:100, 1:500, and 1:1000 were used. 5% brain lysates of a patient with PD and a non‐PD patient served as positive and negative controls and were diluted 1:100. Skin samples of healthy individuals served as additional negative controls.

98 μL of the SAA buffer and 2 μL of the diluted samples were added per well. Each patient sample was tested as a quadruplicate. The plate was sealed with a transparent plastic film and placed in the microplate reader (FLUOstar Omega from BMG Labtech, with software version 5.70 R2).

The fluorescence activity of the samples was measured for 48 h at 37°C. The process alternated between a one‐minute shaking phase at 400 rpm and a 5‐min aggregation phase. Fluorescence values were recorded every 45 min over 65 cycles in relative fluorescence units (rfu). The threshold for positivity was set at 10,000 rfu (corresponding to 3.85% of the maximum fluorescence and to the rounded mean value plus three standard deviations of samples from 40 individuals without a synucleinopathy that were initially measured to establish the assay).

For data analysis, the BMG LABTECH MARS 4.01 R2 software from BMG Labtech was used. The following parameters were evaluated: Lag time: Defined as the time until fluorescence exceeds the threshold of 10,000 rfu. Positive curve: Curve of one replicate in which fluorescence of more than 10,000 rfu is detected. If at least three curves within a quadruplicate were positive, the sample was considered positive. Number of positive curves: Number of curves of a quadruplicate that exceed the threshold of 10,000 rfu. Titer: The titer describes the highest dilution in which a patient sample (quadruplicate) is still considered positive. The examiner was blinded regarding the clinical phenotypes at the time point of analysis of SAA curves.

### 2.3 Statistical Analysis

The statistical analysis was performed using IBM SPSS Statistics version 29.0.1.1 for macOS (IBM, Armonk, NY, USA) and GraphPad Prism version 10.3.1 for macOS (GraphPadSoftware, San Diego, CA, USA).

The Mann–Whitney *U* test (MWU) was used to compare patient groups and subgroups for titer and the number of positive curves. For the analysis of titers, ascending titers were ranked. The unpaired, two‐tailed *t*‐test was used to compare the lag time between groups, as it met the assumptions of normal distribution and interval scaling.

To compare the three groups, suspected brain‐first, body‐first, and iRBD, a one‐way ANOVA without repeated measures was performed for lag time, and the Kruskal–Wallis test was used to compare titers and the number of positive curves. Spearman's correlation was applied for correlation analyses. For all statistical tests, the significance level was set at 0.05.

## 3 Results

### 3.1 Comparison of Different Quantitative Parameters of Dermal SAA

Lag time, the number of positive curves, and the titer were used as quantitative parameters. We found a negative correlation between lag time and titer or the number of positive curves (*r* = −0.455, *p* = 0.0005 (lag time vs. titer), *r* = −0.774, *p* = 6.5 * 10^−12^ (lag time vs. number of positive curves)) and a positive correlation between the titer and the number of positive curves (*r* = 0.546, *p* = 1.9 * 10^−5^). We did not find a correlation between quantitative SAA parameters and clinical data of PD patients, like stage, progress, and duration of disease, MoCA Score, and NMSS, except for a moderate correlation between age at onset and SAA titer (*r* = 0.33, *p* = 0.042). Double testing of our samples showed 66.7% accordance for the number of positive curves. Comparison of positive results with measurements of a previous study using the same samples but slightly different assay conditions resulted in concordant results in 86.7% of samples.

### 3.2 Higher SAA Titers Are Associated With a Later Onset of PD

Titers, lag time, and the number of positive curves of patients with the following parameters were compared: late/early age at onset, autonomic symptoms, cardiovascular/gastrointestinal symptoms, tremor/hypokinetic dominance, rapid/slow progression, RBD symptoms, and high/low NMSS. A higher titer was found in patients with a late onset of disease (> 52 years, *p* = 0.023), and a trend towards a higher titer, more positive curves, and lower lag time was found in PD patients with RBD symptoms but failed to reach significance. Conversely, the aforementioned characteristics did not differ between patients with high, low, or negative values for titer, lag time, or number of positive curves. All healthy controls were negative.

Patients were divided into suspected brain‐ and body‐first subtypes as described above, resulting in 19 brain‐first and 20 body‐first patients. Demographic and clinical details of the groups are summarized in Table 1.

**TABLE 1:** Demographic, clinical, and SAA data of patients with suspected brain−/body‐first PD and iRBD.

	Suspected brain‐first (*n* = 19)	Suspected body‐first (*n* = 20)	iRBD^a^ (*n* = 15)
Mean age in years (SD)	66.42 (9.09)	73.25 (7.17)	62.89 (4.21)
Sex female/male (*n*)	8/11	3/17	1/14
Median duration of disease (range)	14 (5–25)	12 (3–27)	6 (2–20)
Median age at onset (range)	50 (36/−66)	55 (36–81)	53 (47–63)
H&Y^b^ (*n*)	I: 1, II: 6, III: 10, IV: 2	I: 0, II: 7, III: 10, IV: 3	
Median Progress (H&Y/year)	0.182 (0.118–0.500)	0.2 (0.106–1.000)	
Median NMSS^c^ (range)	27 (6–128)	43 (7–169)	5 (1–19)
Median MoCA^d^ (range)	28 (21–30)	25 (17–29)	28 (24–30)
RBD^a^ yes/no (*n*)	3/16	15/5	15 (polysomnographically confirmed)
Symptom at onset Hypokinetic/tremor‐dominant (*n*)	5/14	13/7	
Average lag time (h)	28.0	29.3	25.5
Average number of positive curves	2.9	2.4	2.9
Titers (*n*)	Negative: 8, 1:10: 8, 1:50: 0, 1:100: 1, 1:500: 2, 1:1000: 0	Negative: 4, 1:10: 6, 1:50: 2, 1:100: 3, 1:500: 2, 1:1000: 3	Negative: 1, 1:10: 3, 1:50: 0, 1:100: 2, 1:500: 4, 1:1000: 5

^a^ Isolated; REM sleep behavior disorder.

^b^ Hoehn & Yahr stage.

^c^ Non‐motor symptom scale.

^d^ Montreal cognitive assessment.

The median titer of suspected body‐first (1:50) patients was slightly higher compared to suspected brain‐first patients (1:10, *p* = 0.034); the number of positive curves and the lag time did not differ.

### 3.3 SAA Titers Are Higher in iRBD Patients Compared to PD

**FIGURE 1:**
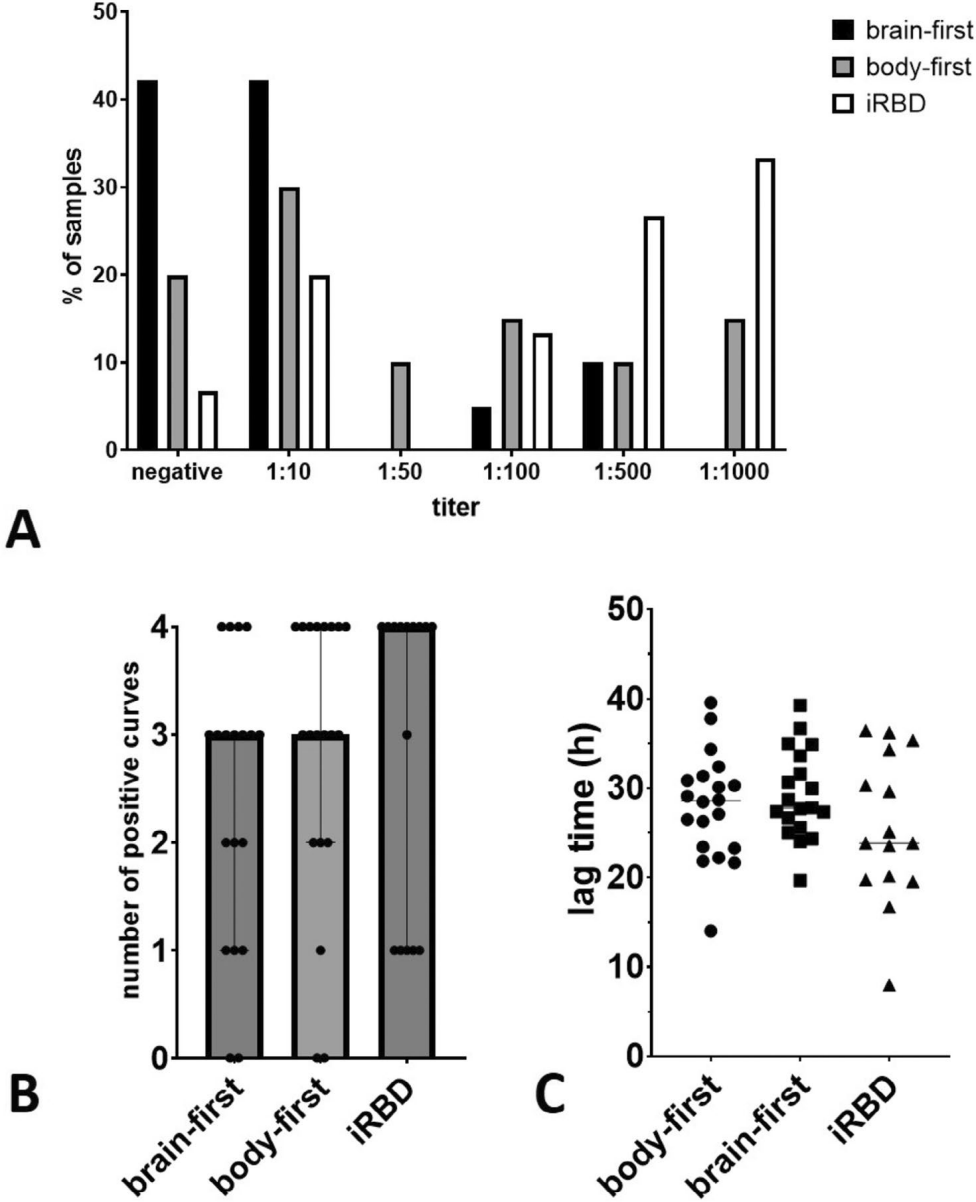
Distribution of quantitative SAA parameters in the subgroups suspected of brain‐first/body‐first PD and iRBD. (A) The distribution of the titers, indicating a larger percentage with lower titers in the brain‐first group. (B) The distribution of the number of positive replicates that did not significantly differ between groups. Bars indicate means, whiskers mark standard deviations. Lag times also did not significantly differ (C).

As the differentiation between brain‐ and body‐first PD primarily refers to the onset or very early stages of disease, and differences may vanish at later stages when a‐syn spreading possibly affects the whole nervous system, we included samples of a cohort of iRBD patients. iRBD is supposed to be a premotor stage of body‐first PD in the majority of cases. In contrast, premotor stages of brain‐first PD are difficult to identify and collect. Therefore, the iRBD samples were compared with the whole PD cohort and the two subgroups (suspected brain‐ and body‐first). The titer significantly differed between PD (median 1:10) and iRBD patients (median 1:500, *p* = 0.002) and between suspected brain‐first (median 1:10) and iRBD patients (*p* < 0.001) and also tended to be higher in iRBD compared to suspected body‐first PD patients (1:50, *p* = 0.07). The number of positive curves and the lag time did not significantly differ between groups. Figure 1 illustrates the distribution of titers, numbers of positive curves, and lag time in the three groups.

## 4 Discussion

In the present study, we aimed at distinguishing between PD patients with features of a suspected brain‐ and body‐first subtype by quantitative SAA parameters. We only found small differences, showing a trend towards higher a‐syn deposition in patients with features of a suspected body‐first subtype. However, when comparing a cohort with a confirmed diagnosis of iRBD, mostly reflecting very early stages of hypothesized body‐first PD or dementia with Lewy bodies, a‐syn deposition was higher compared to PD, especially compared to suspected brain‐first PD. This may indicate early and strong involvement of peripheral autonomic nerve fibers in this subgroup. The low difference between subtypes may be explained by widespread a‐syn pathology at advanced stages of disease or decreased dermal autonomic nerve fibers because of degeneration as a possible confounder.

The three parameters of quantification analyzed in this study did not deliver uniform results, even if they showed correlation. Double testing also showed some variation of results. This reflects the difficulties of quantification of SAA that are caused by different factors like strong effects of even small variances of samples, reagents, and assay conditions, as well as the stochastic, non‐linear reaction of the assay and the lack of standardized calibration [4]. Previous studies aimed at developing quantitative protocols and scores on the basis of dilution series or the use of digital SAA may improve quantification [13, 14].

In a previous study comparing immunohistochemical detection of phospho‐a‐syn in PD patients with and without suspected RBD, we found a higher number of positive cases in those who reported RBD [15]. In another study, the percentage of p‐a‐syn positivity in the skin was higher in RBD patients compared to PD [16]. When applying dermal SAA in cohorts with PD and iRBD, we found a slightly lower lag time and higher rate of positive patients in the iRBD cohort in a recent study [10], indicating that iRBD is associated with abundant a‐syn pathology in the peripheral nervous system. The overall lower number of positive samples in the current study compared to previous studies is explained by the restriction of our current quantitative analysis to one sample per patient.

In addition to iRBD being an indicative symptom of hypothesized body‐first PD, more abundant a‐syn pathology may be caused by a generally more malignant course of disease in patients with an initial diagnosis of iRBD [17, 18]. Indeed, titers of dermal SAA of iRBD patients tended to be higher even in comparison with suspected body‐first PD in our study. This may indicate that iRBD is generally associated with more abundant a‐syn deposition but may also be explained by a less defined cohort of body‐first subtype in the PD group, as the subtype cannot be clearly identified at later stages of disease, and RBD in the PD group was not diagnosed by polysomnography. Furthermore, the iRBD cohort may also contain patients who later develop dementia with Lewy bodies, which are also associated with abundant dermal a‐syn aggregation [19].

Categorization of PD subtypes in our study was based on characteristic features that were reported in the literature [7] but no validated classification is available so far, and different features may have different impacts.

However, quantification of dermal a‐syn by SAA did not reveal any big differences and did not show any bimodal distribution of dermal a‐syn load, arguing in favor of a widespread distribution of a‐syn in autonomic nerve fibers in patients of both subtypes. Those findings are in line with the brain−body‐first hypothesis that assumes spreading of a‐syn pathology during the course of disease [20] and indicate that in a cohort of mixed stages of PD as found in medical centers and hospitals, a‐syn has already widely spread, and dermal SAA does not allow a categorization on the level of single patients. By using immunohistochemical staining of phospho‐a‐syn, a recent study also reported positive findings in patients with and without autonomic abnormalities, but could detect an increase in skin intraneural phospho‐a‐syn load in individuals with cognitive and autonomic abnormalities [21].

Besides the general difficulty of categorization of PD patients into subtypes, especially in advanced stages of disease, and the difficulties of quantification of a‐syn aggregates by SAA, our study has other limitations: Patients were not systematically screened for genetic forms of PD. Samples were stored in our department as biopsies were taken between 2019 and 2022, and even if we did not find any correlation between storage time and a‐syn aggregation, a decrease during storage cannot be excluded. Analysis of several biopsy sites per patient may provide information on spreading of a‐syn in different PD subtypes, and prospective collection of clinical data over a longer time period may allow a better classification of patients. For the determination of the sensitivity of SAA testing in different clinical phenotypes, a larger number of control subjects would be needed. These issues may be addressed in future studies.

In summary, this study provides evidence that iRBD patients have a higher dermal a‐syn load than PD patients that can be measured by quantitative SAA but our data point out that clinical and SAA subgroup differentiation may be difficult in a patient cohort of various stages.

## Author Contributions

Magdalena Vieregge: acquisition and analysis of data and statistical tests. Anastasia Kuzkina: patient recruitment and data acquisition. Annette Janzen: patient recruitment and data acquisition. Wolfgang H. Oertel: patient recruitment and data acquisition. Michael Sommerauer: patient recruitment and data acquisition. Jens Volkmann: patient recruitment and data acquisition. Kathrin Doppler: study concept and design, data analysis, statistical testing, and writing of the draft.

## Funding

This work was supported by German Federal Ministry of Education and Research (BMBF).

## Conflicts of Interest

The authors declare no conflicts of interest.

## Data Availability

The data that support the findings of this study are available from the corresponding author upon reasonable request.
